# Risk of Bias from Inclusion of Currently Diagnosed or Treated Patients in Studies of Depression Screening Tool Accuracy: A Cross-Sectional Analysis of Recently Published Primary Studies and Meta-Analyses

**DOI:** 10.1371/journal.pone.0150067

**Published:** 2016-02-26

**Authors:** Danielle B. Rice, Brett D. Thombs

**Affiliations:** 1 Lady Davis Institute for Medical Research, Jewish General Hospital, Montréal, Québec, Canada; 2 Department of Psychiatry, McGill University, Montréal, Québec, Canada; 3 Department of Epidemiology, Biostatistics, and Occupational Health, McGill University, Montréal, Québec, Canada; 4 Department of Psychology, McGill University, Montréal, Québec, Canada; 5 Department of Medicine, McGill University, Montréal, Québec, Canada; 6 Department of Educational and Counselling Psychology, McGill University, Montréal, Québec, Canada; 7 School of Nursing, McGill University, Montréal, Québec, Canada; Peking Union Medical College Hospital, CHINA

## Abstract

**Background:**

Depression screening can improve upon usual care only if screening tools accurately identify depressed patients who would not otherwise be recognized by healthcare providers. Inclusion of patients already being treated for depression in studies of screening tool accuracy would inflate estimates of screening accuracy and yield. The present study investigated (1) the proportion of primary studies of depression screening tool accuracy that were recently published in journals listed in MEDLINE, which appropriately excluded currently diagnosed or treated patients; and (2) whether recently published meta-analyses identified the inclusion of currently diagnosed or treated patients as a potential source of bias.

**Methods:**

MEDLINE was searched from January 1, 2013 through March 27, 2015 for primary studies and meta-analyses on depression screening tool accuracy.

**Results:**

Only 5 of 89 (5.6%) primary studies excluded currently diagnosed or treated patients from any analyses and only 3 (3.4%) from main analyses. In 3 studies that reported the number of patients excluded due to current treatment, the number of excluded patients was more than twice the number of newly identified depression cases. None of 5 meta-analyses identified the inclusion of currently diagnosed and treated patients as a potential source of bias.

**Conclusions:**

The inclusion of currently diagnosed and treated patients in studies of depression screening tool accuracy is a problem that limits the applicability of research findings for actual clinical practice. Studies are needed that evaluate the diagnostic accuracy of depression screening tools among only untreated patients who would potentially be screened in practice.

## Introduction

Major depression is present in 5–10% of primary care patients, including 10–20% of patients with chronic medical conditions [1, 2]. Effective treatments are available, but approximately half of depressed patients go unrecognized [3]. At the same time, overdiagnosis and overtreatment are common [4, 5]. Among older primary care patients in the United States (US), for example, fewer than 20% of those diagnosed with depression by a physician or prescribed antidepressant medication meet major depression diagnostic criteria [6].

The vast majority of depression care is provided outside of psychiatric settings [7], and depression screening has been proposed as a way to improve identification and management of depression in primary [1, 8] and specialty care settings [9–15]. Depression screening guidelines and policies vary substantially, however. The US Preventive Services Task Force recommends depression screening in primary care settings with integrated, collaborative depression care systems [1]. Accreditation for many healthcare providers in the US requires documentation of depression screening [16], and depression screening is a required component of Medicare’s Annual Wellness Visit [17]. In the United Kingdom (UK), on the other hand, neither the National Institute for Health and Care Excellence [2] nor the UK National Screening Committee [18] recommend routine depression screening. The UK Quality and Outcome Framework incentivized routine depression screening in primary care from 2006 to 2013, but discontinued the program due to disappointing outcomes [8, 19]. In Canada, depression screening was previously recommended in primary care, but in 2013 the Canadian Task Force on Preventative Health Care recommended against it [20]. In their recommendation, the Canadian Task Force raised the concern that existing research may exaggerate the diagnostic accuracy of depression screening tools [21].

For depression screening to improve upon usual care, screening tools must accurately identify patients who are not currently in treatment or seeking treatment and whose depression would not otherwise be recognized by a healthcare provider [20–22]. A 2011 study, however, reported that only 4% of primary studies included in 17 systematic reviews on depression screening tool accuracy appropriately excluded patients currently diagnosed or being treated for depression [21]. Since screening is done to identify previously unrecognized cases, including potentially large numbers of patients already being treated would exaggerate estimates of the accuracy of screening tools and the yield of new cases from screening [21].

It is not known whether more recently published studies have excluded currently diagnosed and treated patients in order to generate results that are more applicable for clinical practice. The objectives of the present study were to investigate (1) the proportion of primary studies of depression screening tool accuracy that were recently published in journals listed in MEDLINE, which appropriately excluded patients with current depression diagnoses or treatment at the time of study enrolment; and (2) whether recent meta-analyses identified the failure to exclude currently diagnosed and treated patients from primary studies as a potential source of bias.

## Methods

### Article Selection

Our objective was not to conduct a systematic review of screening tool accuracy. Rather, it was to evaluate inclusion and exclusion criteria in studies likely to influence future research methods, policy and practice. A recent study found that restricting a search to only MEDLINE for studies of diagnostic test accuracy did not influence summary estimates in meta-analyses [23]. Consistent with this, studies of depression screening tool accuracy that were published in journals not listed in MEDLINE would not be likely to substantively influence future research methods, policy, or practice. Thus, we limited our search to MEDLINE. We searched MEDLINE (PubMed interface) on March 27, 2015 for primary studies and meta-analyses published in 2013 or later that evaluated the diagnostic accuracy of depression screening using the search terms (depress* AND sensitivity AND specificity), restricted to title or abstract. We included studies published in 2013 or later to obtain recent studies that reflect current practices, which were published long enough after the 2011 review on this topic [21] to incorporate recommendations.

Eligible primary studies were published in any language and reported the accuracy of one or more depression screening tools compared to a diagnosis of depression based on clinician interview or a validated diagnostic interview. Studies were excluded if the diagnostic reference standard was based on a chart diagnosis or a score above a threshold on another self-report measure or rating scale. Studies that included only patients in mental health treatment were also excluded since screening is not meant to be applied to patients already receiving treatment.

Eligible meta-analyses: (1) included a systematic review of the literature using at least one electronic database; (2) statistically combined results from ≥ 2 primary studies; and (3) reported measures of diagnostic accuracy (e.g., sensitivity, specificity) for one or more depression screening tools compared to depression diagnoses. We excluded systematic reviews without meta-analyses because commonly used screening tools are more likely to be included in meta-analyses. Publications that included meta-analyses of the diagnostic accuracy of screening tools for depression, as well as for other disorders, were included if results were presented separately for depression.

Citations were uploaded directly from PubMed into the systematic review manager DistillerSR (Evidence Partners, Ottawa, Canada), which was used for all coding procedures, including tracking the review process and data extraction. Two investigators independently reviewed primary studies for eligibility. If either reviewer deemed a study potentially eligible based on title and abstract review, full text review was conducted. Disagreements between reviewers after full-text review were resolved by consensus.

### Data Extraction and Classification

One investigator extracted data from each included study with independent validation by a second reviewer. For each primary study, we extracted the screening tool(s) evaluated, reference standard, study population, number of patients, number of depression cases, and whether the study excluded patients currently diagnosed or treated for depression. Primary studies were classified as having excluded patients with current depression diagnosis or treatment if the study authors specifically indicated this in the exclusion criteria. Studies were classified as having included currently diagnosed or treated patients if the study did not specifically indicate that such patients were excluded. For each meta-analysis, we extracted author, publication year, journal, and 2014 journal impact factor. For publications that included meta-analyses of diagnostic accuracy and other measurement characteristics (e.g., reliability) only diagnostic accuracy results were extracted. For each meta-analysis, investigators recorded whether the authors identified the inclusion of currently diagnosed or treated patients in primary studies as a possible source of bias.

## Results

### Article Selection

**Fig 1:**
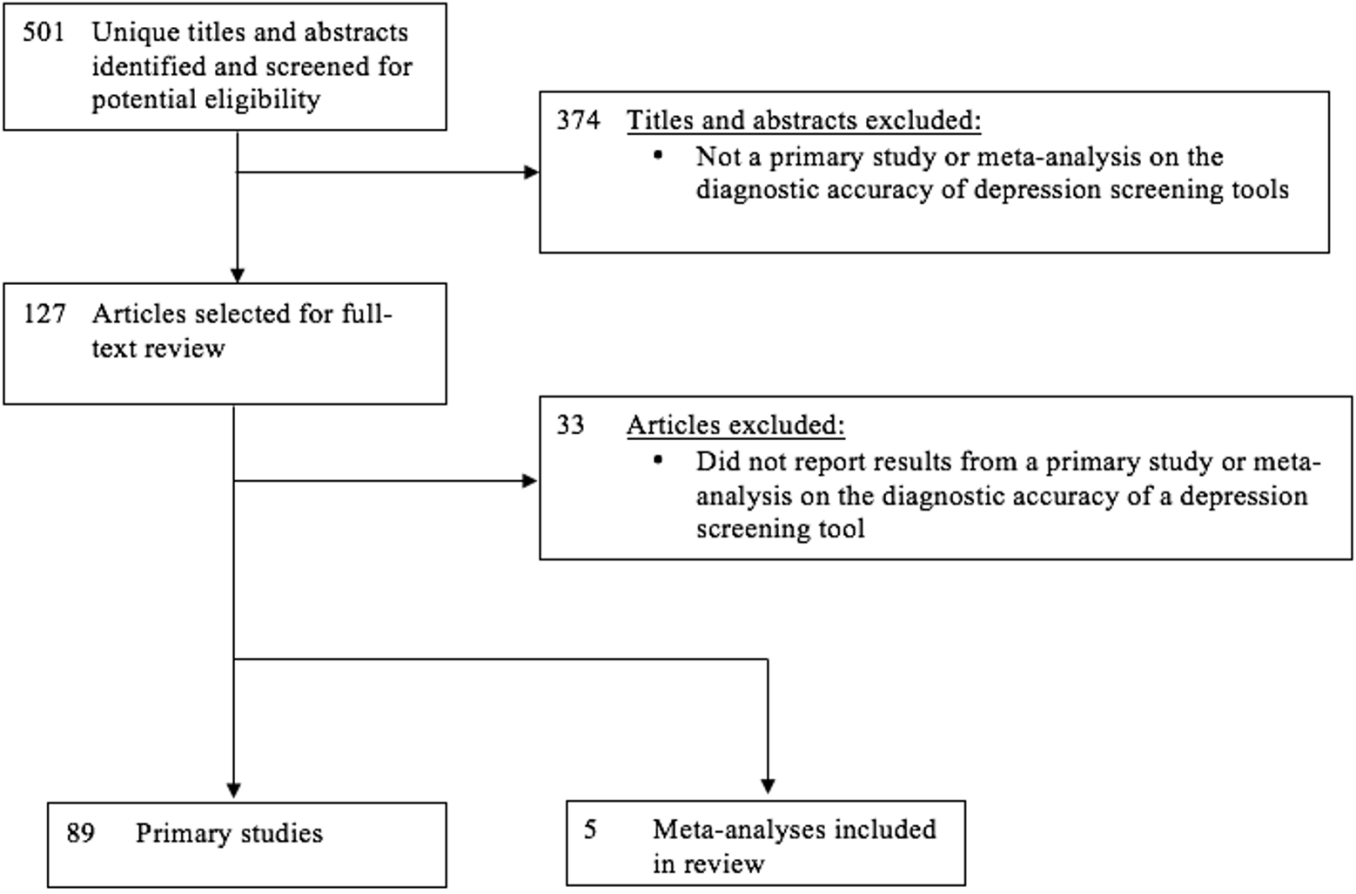
Flow Diagram of Selection of Primary Studies and Meta-analyses that Evaluated the Diagnostic Accuracy of Depression Screening Tools.

The database search yielded 501 unique titles and abstracts. Of these, 374 were excluded after title and abstract review and 33 after full-text review because they did not report results from a primary study or meta-analysis that evaluated the diagnostic accuracy of a depression screening tool, leaving 89 eligible primary studies and 5 eligible meta-analyses (Fig 1). The 89 primary studies included sample sizes from 34 to 42,676 (median = 224) and number of depression cases from 5 to 3,115 (median = 37). The majority of studies were from Europe (28%), Asia (24%) or North America (19%). Primary study characteristics are shown in S1 Appendix. Characteristics of included meta-analyses are shown in Table 1.

**Table 1:** Characteristics of Included Meta-Analyses.

First Author, Year of Publication	Journal (2014 Impact Factor)	Focus of Meta-Analysis	Screening Tool(s) Meta-Analyzed	Range of Years of Publication of Included Primary Studies
Manea, 2015	Gen Hosp Psychiatry (2.6)	PHQ-9 algorithm scoring in any setting	PHQ-9	2001–2013
Stockings, 2015	J Affect Disord (3.4)	Screening in children and adolescents	BDI, CESD,CDI	1984–2012
Meader, 2014	J Neurol Neurosurg Psychiatry (6.8)	Screening tools in poststroke patients	BDI or BDI-II, CESD, GDS-15, HADS-D, HADS-T, PHQ-2, PHQ-9	1988–2012
Tsai, 2014	JAIDS (4.6)	Screening tools in HIV-positive adults in Africa	CESD	2008–2012
Tsai, 2013	PLoS One (3.2)	Screening tools in pregnancy or postpartum in Africa	EPDS	1998–2011

BDI = Beck Depression Inventory; CDI = Children’s Depression Inventory; CESD = Center for Epidemiologic Studies Depression Scale; EPDS = Edinburgh Postnatal Depression Scale; GDS = Geriatric Depression Scale; HADS-D = Hospital Anxiety and Depression Scale–depression subscale; HADS-T = Hospital Anxiety and Depression Scale—total score; PHQ-2 = Patient Health Questionnaire-2; PHQ-9 = Patient Health Questionnaire-9.

#### Exclusion of Currently Diagnosed and Treated Patients

Only 5 of 89 primary studies (5.6%) excluded patients with a current diagnosis or currently being treated for depression at the time of study enrolment from any analyses [24–28], and only 3 (3.4%) excluded these patients from the main study analyses [24, 25, 26].

There were 3 studies that reported the number of patients excluded for current depression diagnosis or treatment [24–26]. In a study of coronary heart disease patients [24], 65 of 803 (8.1%) otherwise eligible patients were excluded due to current depression diagnosis or treatment. Of the remaining 730 patients (after removal of 8 patients with missing data), 32 (4.4%) were newly diagnosed with a depressive disorder. In a second study, of women with breast or gynaecological cancer [25], 28 of 100 women recruited (28.0%) were excluded due to existing treatment, and 13 of 72 untreated patients (18.1%) were newly diagnosed with a depressive disorder. The third study enrolled 152 Canadian patients with multiple sclerosis [26]. There were 20 patients diagnosed with major depression, but 15 were already being treated for depression at the time of study enrolment.

None of the 5 meta-analyses identified the inclusion of patients currently diagnosed or currently treated for depression in primary studies as a potential source of bias.

## Discussion

The main finding of this study was that fewer than 6% of primary studies on the diagnostic accuracy of depression screening tools published since 2013 excluded patients with a current diagnosis or treatment for depression. Only 3% of studies excluded these patients from main study analyses. None of 5 included meta-analyses identified the inclusion of currently diagnosed and treated patients as a potential source of bias. These results are similar to findings from a 2011 study [21], which first identified this problem. In that study, only 8 of 197 (4%) primary studies in 17 systematic reviews appropriately excluded already diagnosed and treated patients, and none of the systematic reviews mentioned this problem as a potential source of bias.

It is not known to what extent including currently diagnosed and treated patients in primary studies exaggerates the yield of new depression cases and estimates of accuracy compared to what would occur in practice. A previous study [21] estimated that in a primary care population with 10% major depression prevalence, if half of depressed patients were already receiving treatment [3], then properly excluding already diagnosed and treated patients from screening accuracy studies would reduce the positive predictive value (proportion of positive screens that are true cases), from 27% to 14%, even if sensitivity were only exaggerated by 10%.

We identified only 3 studies [25, 26, 29], although none in primary care settings, that have analysed depression screening tool accuracy with and without patients currently diagnosed or receiving treatment, including 2 primary studies from the present study [25, 26]. One study [25], which included 100 female cancer patients (26 with depressive disorders), evaluated the diagnostic accuracy of the Centre for Epidemiological Studies Depression Scale and Hospital Anxiety and Depression Scale. For both screening tools, sensitivity and positive predictive value were reduced by approximately 10% when already-treated patients were excluded. In a second study [29], which involved 113 women with breast cancer, excluding already-treated patients did not change sensitivity, but reduced positive predictive value from 21% to 7%. A third study [26] reported data on 152 multiple sclerosis patients, and 15 of the 20 patients diagnosed with depression in the study were receiving treatment for depression at the time of study enrolment. These studies all involved very small numbers of untreated patients with major depression. Nonetheless, results from these studies are generally consistent with prior estimates [22] and suggest that inclusion of currently diagnosed and treated patients may substantially exaggerate estimates of both the accuracy of depression screening tools and the number of patients who would be newly identified as depressed compared to clinical practice, where only previously unidentified, non-treated patients are screened.

In clinical practice, depression symptom questionnaires, or screening tools, are used by healthcare providers for a number of purposes, including screening to detect previously unidentified cases, tracking treatment progress, or detecting relapse, for instance. For the purpose of screening, however, they are only useful to the extent that they distinguish between disordered and non-disordered states that are not otherwise identified [30]. The 3 primary studies that reported the number of patients who were excluded due to existing depression treatment at the time of study enrolment [24–26] found that more than twice as many patients were excluded for this reason than the number of new cases identified with a screening tool. Thus, it is likely that many of the 94% of primary studies that did not exclude these patients also included large numbers of already-treated patients. This conclusion would be consistent with the high rates of existing depression treatment in the general population and among patients in medical settings. A recent study, for instance, reported that 7% of 26,800 randomly sampled Europeans from 27 countries had used antidepressants in the last 12 months [31]. A US general population survey found a 10% prevalence of current antidepressant use among adults and reported that this was long-term use (at least 24 months) in two-thirds of cases [32]. A systematic review of antidepressants among acute coronary syndrome (ACS) patients found that 10–15% of patients assessed between 2000 and 2005 were prescribed or using antidepressants within 12 months of the index ACS [33]. Similarly, administrative data from Ontario, Canada showed that the rate of antidepressant prescriptions within 6 months of an acute myocardial infarction doubled from 8% in 1993 to 16% in 2002 among patients age 65 and older [34].

Systematic reviews on the effectiveness of depression screening have differed in the studies they included and the conclusions drawn. A systematic review done in conjunction with the 2013 Canadian Task Force on Preventive Health Care depression screening guideline [20] did not identify any eligible randomized controlled trials (RCTs) [35]. A 2008 Cochrane systematic review included 5 RCTs that met some criteria for a depression screening trial and reported that depression screening did not reduce depressive symptoms [36]. In contrast, a systematic review done as part of the 2009 US Preventive Services Task Force guideline [1] included 9 RCTs and concluded that depression screening benefitted patients when provided in the context of staff-assisted collaborative care [37]. That review has been criticized, however, because the main trials used to support the claim that screening benefitted patients were trials of collaborative depression treatment that required a diagnosis of depression to enrol [8, 38]. None of the trials in the Cochrane review or the US Preventive Services Task Force review randomized patients prior to screening, excluded currently treated patients, and provided similar depression treatment to patients identified as depressed through screening and patients identified through usual care [8, 38]. The results of the present study emphasize that it should not be assumed that depression screening programs would accurately identify and successfully treat otherwise unrecognized patients without evidence from a properly designed, well-conducted randomized controlled trial to demonstrate this.

A possible limitation of the present study was that we searched only the MEDLINE database for eligible studies. However, including only MEDLINE for searches of studies of diagnostic test accuracy have been shown to not influence summary estimates in meta-analyses [23]. Thus, it is not likely that our main results would have changed if other databases had been searched. An additional limitation is that only a few studies with a small number of depression cases have presented information on how accuracy estimates are influenced by the inclusion versus exclusion of already diagnosed and treated patients. Thus, we could not determine with precision the effect of inappropriate inclusion of currently diagnosed and treated patients on accuracy estimates. However, many studies from other areas of research have reported that the inclusion of established cases among examined cohorts inflates assessments of diagnostic test accuracy [39]. Individual patient data meta-analysis is an approach that may be able to provide stable estimates of diagnostic accuracy parameters by excluding already treated patients in studies of depression screening tool accuracy [40]. A final limitation, although unlikely, is the possibility that already diagnosed and treated patients could have been excluded from primary studies, but that authors of the primary studies did not report this as an exclusion criterion.

In summary, we found that fewer than 6% of primary studies on the diagnostic accuracy of depression screening tools published since 2013 appropriately excluded patients currently diagnosed or undergoing treatment for depression and that recent meta-analyses have neglected this issue as a potential source of bias. Existing evidence on the accuracy and case yield of depression screening tools may substantially overestimate their utility in clinical practice. Well-designed studies that exclude patients currently diagnosed or treated for depression are needed to generate realistic estimates of accuracy that reflect what would be achieved in clinical practice. Although depression symptom questionnaires are used for a variety of purposes, including follow-up assessment of patients receiving treatment, studies that seek to evaluate their accuracy for identifying patients with previously unrecognized depression must exclude these patients.

## Supporting Information

S1 Appendix Primary Studies of the Diagnostic Accuracy of Depression Screening Tools. Characteristics of included primary studies, including first author and year published, journal, country, population, number of participants, number of depression cases, diagnostic criterion, screening tool, and inclusion or exclusion of currently diagnosed and treated patients.
(DOCX)

S1 Checklist PRISMA Checklist. Table that indicates where in manuscript all elements of the PRISMA Checklist can be found.
(DOC)

## References

[1] U.S. Preventive Services Task Force. Screening for depression in adults: U.S. Preventive Services Task Force recommendation statement. Ann Intern Med. 2009;151:784–92. 10.7326/0003-4819-151-11-200912010-00006 19949144

[2] National Collaborating Center for Mental Health. The NICE guideline on the management and treatment of depression in adults (Updated edition) United Kingdom: National Institute for Health and Clinical Excellence; 2010.

[3] Mitchell AJ, Vaze A, Rao S. Clinical diagnosis of depression in primary care: A meta-analysis. Lancet. 2009;374:609–19. 10.1016/S0140-6736(09)60879-5 19640579

[4] Mojtabai R, Olfson M. Proportion of antidepressants prescribed without a psychiatric diagnosis is growing. Health Aff. 2011;30:1434–42. 10.1377/hlthaff.2010.1024 21821561

[5] Mojtabai R. Clinician-identified depression in community settings: Concordance with structured interview diagnoses. Psychother Psychosom. 2013;82:161–69. 10.1159/000345968 23548817

[6] Mojtabai R. Diagnosing depression in older adults in primary care. NEJM. 2014;370:1180–82. 10.1056/NEJMp1311047 24670164

[7] Meng X, D'Arcy C, Tempier R. Trends in psychotropic use in Saskatchewan from 1983 to 2007. Can J Psychiatry. 2013;58:426–31. 23870725 10.1177/070674371305800708

[8] Thombs BD, Ziegelstein RC. Does depression screening improve depression outcomes in primary care? BMJ. 2014;348:g1253 10.1136/bmj.g1253 24496211

[9] National Institute for Clinical Excellence. Guideline on cancer services: Improving supportive and palliative care for adults with cancer United Kingdom: National Institute for Health and Clinical Excellence; 2004.

[10] National Comprehensive Cancer Network. Distress management. NCCN clinical practice guidelines in oncology National Comprehensive Cancer Network; 2008. 10.6004/jnccn.2019.0048 PMC6907687 31590149

[11] Holland JC, Andersen B, Breitbart WS, Buchmann LO, Compas B, Deshields TL, et al Distress management. J Natl Compr Canc Netw. 2013;11:190–209. 23411386 10.6004/jnccn.2013.0027

[12] Lichtman JH, Bigger JT Jr, Blumenthal JA, Frasue-Smith N, Kaufmann PG, Lespérance F, et al Depression and coronary heart disease: recommendations for screening, referral, and treatment: a science advisory from the American Heart Association Prevention Committee of the Council on Cardiovascular Nursing, Council on Clinical Cardiology, Council on Epidemiology and Prevention, and Interdisciplinary Council on Quality of Care and Outcomes Research: endorsed by the American Psychiatric Association. Circulation. 2008;118:1768–75. 10.1161/CIRCULATIONAHA.108.190769 18824640

[13] Colquhoun DM, Bunker SJ, Clarke DM, Glozier N, Hare DL, Hickie IB, et al Screening, referral and treatment for depression in patients with coronary heart disease. Med J Aust. 2013;198:483–84. 23682890 10.5694/mja13.10153

[14] Canadian Diabetes Association Clinical Practice Guidelines Expert Committee of the Canadian Diabetes Advisory Board. Canadian Diabetes Association 2013 clinical practice guidelines for the prevention and management of diabetes in Canada. Can J Diabetes. 2013;37:S1–S212. 10.1016/j.jcjd.2013.01.009 24070926

[15] Eskes GA, Lanctot KL, Herrmann N, Lindsay P, Bayley M, Bouvier L et al Canadian stroke best practice recommendations: Mood, cognition and fatigue following stroke practice guidelines, update 2015. Int J Stroke. 2015 [Epub ahead of print]. 10.1111/ijs.12557 26121596

[16] NCQA level 3 PCMH recognition requirements compared to 2011 Joint Commission standards and EPs. Available: www.jointcommission.org/assets/1/18/PCMH-NCQA_crosswalk-final_June_2011.pdf (accessed 2012 August 21).

[17] Centers for Medicare and Medicaid Services. Medicare Program; Payment policies under the physician fee schedule and other revisions to part B for CY 2011 November 29, 2010. Available: www.federalregister.gov/articles/2010/11/29/2010-27969/medicare-program-payment-policies-under-the-physician-fee-schedule-and-otherrevisions-to-part-b-for#h-117 (accessed 2015 August 21).

[18] Allaby M. Screening for depression: A report for the UK National Screening Committee (Revised report) United Kingdom: UK National Screening Committee; 2010.

[19] Burton C, Simpson C, Anderson N. Diagnosis and treatment of depression following routine screening in patients with coronary heart disease or diabetes: a database cohort study. Psychol Med. 2013;43:529–37. 10.1017/S0033291712001481 22804849

[20] Joffres M, Jaramillo A, Dickinson J, Lewin G, Pottie K, Shaw E, et al Recommendations on screening for depression in adults. CMAJ. 2013;185:775–82. 10.1503/cmaj.130403 23670157 PMC3680556

[21] Thombs BD, Arthurs E, El-Baalbaki G, Meijer A, Ziegelstein RC, Steele R. Risk of bias from inclusion of already diagnosed or treated patients in diagnostic accuracy studies of depression screening tools: A systematic review. BMJ. 2011;343:d4825 10.1136/bmj.d4825 21852353 PMC3191850

[22] Thombs BD, Coyne JC, Cuijpers P. Rethinking recommendations for screening for depression in primary care. CMAJ. 2012;184:413–18. 10.1503/cmaj.111035 21930744 PMC3291670

[23] van Enst WA, Scholten RJ, Whiting P, Zwinderman AH, Hooft L. Meta-epidemiologic analysis indicates that MEDLINE searches are sufficient for diagnostic test accuracy systematic reviews. J Clin Epidemiol. 2014;67:1192–99. 10.1016/j.jclinepi.2014.05.008 24996667

[24] Haddad M, Walters P, Philips R, Tsakok J, Williams P, Mann A, et al Detecting depression in patients with coronary heart disease: a diagnostic evaluation of the PHQ-9 and HADS-D in primary care, findings from the UPBEAT-UK study. PLOS One. 2013;8:e78493 10.1371/journal.pone.0078493 24130903 PMC3795055

[25] Stafford L, Judd F, Gibson P, Komiti A, Quinn M, Mann GB. Comparison of the Hospital Anxiety and Depression Scale and the Center for Epidemiological Studies Depression Scale for detecting depression in women with breast or gynaecological cancer. Gen Hosp Psychiatry. 2014;36:74–80. 10.1016/j.genhosppsych.2013.08.010 24200105

[26] Patten SB, Burton JM, Fiest KM, Wiebe S, Bulloch AG, Koch M, et al Validity of four screening scales for major depression in MS. Mult Scler. 2015;21:1064–71. 10.1177/1352458514559297 25583846

[27] Xie Z, Lu X, Hu Y, Ma W, Xie H, Lin K, et al Development and validation of the Geriatric Depression Inventory in Chinese culture. Int Psychogeriatr. 2015;23:1–7. 10.1017/S1041610215000162 25703925

[28] Yang Y, Ding R, Hu D, Zhang F, Sheng L. Reliability and validity of a Chinese version of the HADS for screening depression and anxiety in psycho-cardiological outpatients. Compr Psychiatry. 2014;55:215–20. 10.1016/j.comppsych.2013.08.012 24199886

[29] Coyne JC, Palmer SC, Shapiro PJ, Thompson R, DeMichele A. Distress, psychiatric morbidity, and prescriptions for psychotropic medication in a breast cancer waiting room sample. Gen Hosp Psychiatry. 2004;26:121–28. 15038929 10.1016/j.genhosppsych.2003.08.012

[30] Jaeschke R, Guyatt G, Sackett DL. Users' guides to the medical literature. III. How to use an article about a diagnostic test. Are the results of the study valid? Evidence-based Medicine Working Group. JAMA. 1994;271:389–91. 8283589 10.1001/jama.271.5.389

[31] Lewer D, O’Reilly C, Mojtabai R, Evans-Lacko S. Antidepressant use in 27 European countries: associations with sociodemographic, cultural and economic factors. Br J Psychiatry. 2015 [Epub ahead of print]. 10.1192/bjp.bp.114.156786 26159603

[32] Mojtabai R, Olfson M. National trends in long-term use of antidepressant medications: results from the U.S. National Health and Nutrition Examination Survey. J Clin Psychiatry. 2014;75:169–77. 10.4088/JCP.13m08443 24345349

[33] Czarny MJ, Arthurs E, Coffie DF, Smith C, Steele RJ, Ziegelstein RC, et al Prevalence of antidepressant prescription for use in patients with acute coronary syndrome: A systematic review. PLOS One. 2011;6:e27671 10.1371/journal.pone.0027671 22132126 PMC3222644

[34] Benazon NR, Mamdani MM, Coyne JC. Trends in the prescribing of antidepressants following acute myocardial infarction, 1993–2002. Psychosom Med. 2005;67:916–20. 16314596 10.1097/01.psy.0000188399.80167.aa

[35] Keshavarz H, Fitzpatrick-Lewis D, Streiner DL, Rice M, Ali U, Shannon HS, et al Screening for depression: a systematic review and meta-analysis. CMAJ Open. 2014;1:e159–67. 10.9778/cmajo.20130030 PMC3986010 25077118

[36] Gilbody SD, Sheldon TD, House AD. Screening and case-finding instruments for depression: a meta-analysis. CMAJ. 2008;178:997–03. 10.1503/cmaj.070281 18390942 PMC2276549

[37] O'Connor EA, Whitlock EP, Beil TL, Gaynes BN. Screening for depression in adult patients in primary care settings: a systematic evidence review. Ann Intern Med. 2009;151:793–03. 10.7326/0003-4819-151-11-200912010-00007 19949145

[38] Thombs BD, Ziegelstein RC, Roseman M, Kloda LA, Ioannidis JPA. Update and re-analysis of the United States Preventive Services Task Force systematic review on screening for depression in primary care. BMC Med. 2014;12:13 10.1186/1741-7015-12-13 24472580 PMC3922694

[39] Whiting P, Rutjes AW, Reitsma JB, Glas AS, Bossuyt PM, Kleijnen J. Sources of variation and bias in studies of diagnostic accuracy: A systematic review. Ann Intern Med. 2004;140:189–02. 14757617 10.7326/0003-4819-140-3-200402030-00010

[40] Thombs BD, Benedetti A, Kloda LA, Levis B, Nicolau I, Cuijpers P, et al The diagnostic accuracy of the Patient Health Questionnaire-2 (PHQ-2), Patient Health Questionnaire-8 (PHQ-8), and Patient Health Questionnaire-9 (PHQ-9) for detecting major depression: protocol for a systematic review and individual patient data meta-analyses. Syst Rev. 2014;3:124 10.1186/2046-4053-3-124 25348422 PMC4218786

