# Supplementary material for: Risk of Bias from Inclusion of Currently Diagnosed or Treated Patients in Studies of Depression Screening Tool Accuracy: A Cross-Sectional Analysis of Recently Published Primary Studies and Meta-Analyses
Source: PLoS One. 2016 Feb 26;11(2):e0150067. doi: 10.1371/journal.pone.0150067 (PMC4769287; doi:10.1371/journal.pone.0150067)
Supplement: S1 Appendix — Characteristics of included primary studies, including first author and year published, journal, country, population, number of participants, number of depression cases, diagnostic criterion, screening tool, and inclusion or exclusion of currently diagnosed and treated patients. (DOCX) [file pone.0150067.s001.docx]

**S1 Appendix. Primary Studies of the Diagnostic Accuracy of Depression Screening Tools.**

| **First Author,**  **Year** | **Journal** | **Country** | **Population** | **N Screened and Interviewed** | **N Major Depression Cases** | **Diagnostic Criterion** | **Screening Tool(s)** | **Currently Diagnosed or Treated Patients Included or Excluded** |
| --- | --- | --- | --- | --- | --- | --- | --- | --- |
| Akena,  2013 | AIDS Care | Uganda | HIV patients | 368 | 64 | MINI | CESD; K-10; PHQ-9 | Included |
| Ali,  2013 | J Assoc Physicians India | India | Type 2 diabetes patients and healthy controls | 122 | 33 | MINI | BDI | Included |
| Allgaier,  2013 | Int J Geriatr Psychiatry | Germany | Nursing home patients | 92 | 26 | SCID | GDS-15; WHO-5 | Included |
| Bailon,  2013 | Age Ageing | UK | Parkinson's disease patients | 120 | 19 | SCAN | GDS-15; Two-item screener | Included |
| Brunoni,  2013 | Int J Gynaecol Obstet | Brazil | Pregnant women | 543 | 60 | PRIME-MD | IDS-SR | Included |
| Cassin,  2013 | Psychosomatics | Canada | Bariatric surgery candidates | 275 | 10 | MINI | PHQ-9 | Included |
| Chagas,  2013 | Age Ageing | Brazil | Parkinson's disease patients | 84 | 19 | SCID-IV | GDS-15; PHQ-9; ZSDS | Included |
| Chen,  2013 | Asia Pac Psychiatry | China | Primary care patients | 280 | NR | SCID | PHQ-9 | Included |
| Dahm,  2013 | J Affect Disord | Australia | Traumatic brain injury patients | 123 | 47 | SCID | DASS; DASS-21; HADS | Included |
| Fine,  2013 | J Affect Disord | USA | National Guard soldiers | 498 | 121 | SCID-IV | PHQ-9 | Included |
| Ganguly,  2013 | J Adolesc Health | India | Adolescents in schools | 233 | 31 | K-SADS | BDI; PHQ-9 | Included |
| Gelaye,  2013 | Psychiatry Res | Ethiopia | Hospital outpatients | 363 | 46 | SCAN | PHQ-9 | Included |
| Gibbons,  2013 | J Clin Psychiatry | USA | Psychiatry outpatients and general population | 656 | 134 | SCID-IV | PHQ-9 | Included |
| Haddad,  2013 | PLoS One | UK | Coronary heart disease patients | 730 | 32 | CIS-R | HADS; PHQ-9 | Excluded |
| Head,  2013 | Psychol Med | UK | Older-aged civil servants | 274 | 12 | CIS-R | CESD; GHQ | Included |
| Inagaki,  2013 | Gen Hosp Psychiatry | Japan | Primary care patients | 104 | 37 | MINI | PHQ-2; PHQ-9 | Included |
| Juliao,  2013 | J Palliat Med | Portugal | Palliative care patients | 63 | 24 | DSM-IV diagnosis | TLP | Included |
| Kang,  2013 | J Affect Disord | South Korea | Post-stroke patients | 423 | 108 | MINI | BDI; HADS | Included |
| Kessler,  2013 | Psychol Med | USA | Primary care patients | 206 | 41 | SCID | CIDI-Screening Scale | Included |
| Le Strat,  2013 | Compr Psychiatry | USA | General population | 42,676 | 3,115 | AUDADIS-IV | Single-item screener | Included |
| Massoudi,  2013 | J Affect Disord | Sweden | Postnatal fathers | 262 | 29 | PRIME-MD | EPDS | Included |
| Ober,  2013 | Drug Alcohol Rev | Australia | Indigenous inmates | 379 | 54 | CIDI | IRIS | Included |
| Olagunju,  2013 | Psychooncology | Nigeria | Cancer patients | 200 | 55 | SCAN | CESD | Included |
| Orive,  2013 | Assessment | Spain | Medical outpatients | 167 | 89 | PRIME-MD | BDI-PC; DMI-10; DMI-18; HADS; PHQ-9 | Included |
| Picardi,  2013 | Clin Pract Epidemiol Ment Health | Italy | Primary care patients | 212 | 61 | SCID | PC-SAD | Included |
| Pereira,  2013 | Arch Womens Ment Health | Portugal | Pregnant and postpartum women | 453 | 18 | DSM-IV | BDI-II; PDSS | Included |
| Rochat,  2013 | Arch Womens Ment Health | South Africa | Pregnant women | 109 | 51 | SCID | EPDS | Included |
| Rooney,  2013 | Neuro Oncol | UK | Cerebral glioma patients | 155 | 21 | SCID | DT; HADS-D; PHQ-9 | Included |
| Santos,  2013 | Cad Saude Pública | Brazil | Adults 20 years and older from general population | 447 | 40 | MINI | PHQ-9 | Included |
| Sato,  2013 | Mod Rheumatol | Japan | Rheumatoid arthritis patients | 162 | 11 | MINI | CESD; two-item screener | Included |
| Sung,  2013 | Asia Pac Psychiatry | Singapore | Primary care patients | 400 | 36 | MINI | PHQ-9; QIDS-SR16 | Included |
| Taylor,  2013 | J Palliat Med | UK | Palliative care patients | 50 | 10 | MINI | Single-item screener | Included |
| Töreki,  2013 | Midwifery | Hungary | Pregnant women | 219 | 22 | SCID-IV | EPDS | Included |
| Tran,  2013 | BMC Psychiatry | Vietnam | Rural women with young children | 221 | 26 | SCID-IV | DASS-21 | Included |
| Twist,  2013 | Psychosom Med | UK | Type 2 diabetes patients | 368 | 84 | SCAN | PHQ-9 | Included |
| Vilagut,  2013 | Value Health | Belgium; France; Germany; Italy; Netherland; Spain | General population | 21,425 | 321 | CIDI | SF-12 MCS | Included |
| Wagner,  2013 | J Child Neurol | USA | Youth with epilepsy | 87 | 5 | SADS | NDDIE-Youth | Included |
| Wongpakaran,  2013 | J Clin Med Res | Thailand | Geriatric outpatients and long-term care home residents | 237 | 91 | MINI | GDS-15 | Included |
| Zhang,  2013 | J Affect Disord | China | Type 2 diabetes patients | 99 | 23 | MINI | PHQ-9 | Included |
| Zhang,  2013 | Asia Pac Psychiatry | China | College students | 959 | 84 | SCID-IV | PHQ-9 | Included |
| Zis,  2013 | Epilepsy Behav | Greece | Epilepsy patients | 101 | 22 | MINI | NDDI-E | Included |
| Almeida,  2014 | PLoS One | Australia | Indigenous Australians aged 45 years or older | 235 | 18 | DSM-IV diagnosis | KICA-dep | Included |
| Alvaredo-Esquivel,  2014 | J Clin Med Res | Mexico | Pregnant women | 158 | 37 | DSM-IV diagnosis | EPDS | Included |
| Alvaredo-Esquivel, 2014 | Clin Pract Epidemiol Ment Health | Mexico | Pregnant teenagers | 120 | 27 | DSM-IV diagnosis | EPDS | Included |
| Baillon,  2014 | Int J Geriatr Psychiatry | UK | Parkinson's Disease | 120 | 19 | SCAN | EPDS | Included |
| Chan,  2014 | Int J Rheum Dis | China | Axial spondyloarthritis patients | 160 | 17 | SCID | HADS | Included |
| Choi,  2014 | Spine J | USA | Chronic spinal disorder patients | 542 | 331 | SCID-IV | BDI; SF-36 MCS; PHQ-9 | Included |
| Cholera,  2014 | J Affect Disord | South Africa | Primary care patients in high HIV burden setting | 397 | 47 | MINI | PHQ-9 | Included |
| de Figueiredo,  2014 | Arch Womens Ment Health | Brazil | Postpartum women | 199 | 90 | SCID | EPDS | Included |
| de Silva,  2014 | Ceylon Med J | Sri Lanka | Psychiatry outpatients and community | 151 | 76 | SCID | CESD | Included |
| Fiest,  2014 | Epilepsia | Canada | Epilepsy patients | 185 | 27 | SCID | HADS; PHQ-9 | Included |
| Gelaye,  2014 | Ann Epidemiol | Ethiopia | Hospital outpatients | 926 | 162 | CIDI | PHQ-9 | Included |
| Hanwella,  2014 | Depress Res Treat | Sri Lanka | Psychiatry outpatients and community | 151 | 76 | SCID-IV | PHQ-9 | Included |
| Hobkirk,  2014 | Psychol Assess | USA | HIV-positive patients | 1583 | 227 | CIDI | BDI-II | Included |
| Hsu,  2014 | Int J Nurs Stud | Taiwan | Type 2 diabetes patients | 212 | 36 | DSM-IV | BDI-II; CUDOS | Included |
| Husain,  2014 | J Immigr Minor Health | UK | Pregnant women of Pakistani origin | 237 | 63 | SCAN | EPDS | Included |
| Law,  2014 | J Clin Sleep Med | Australia | Suspected obstructive sleep apnea patients | 101 | 30 | MINI | BDI-FS; HADS | Included |
| Lees,  2014 | Cerebrovasc Dis | UK | Stroke patients | 69 | 12 | MINI | DISCs; HADS | Included |
| Lino,  2014 | PLoS One | Brazil | Primary care patients aged 59 or older | 142 | 37 | SCID-IV diagnosis | PHQ-2 | Included |
| Makanjuola,  2014 | Gen Hosp Psychiatry | Nigeria | Primary care patients | 1590 | NR | CIDI | GHQ-12; K-6 | Included |
| Matijasevich,2014 | BMC Psychiatry | Brazil | General population | 447 | 40 | MINI | EPDS | Included |
| Moullec,  2014 | Eur J Prev Cardiol | Canada | Cardiac outpatients | 750 | 42 | PRIME-MD | BDI-II | Included |
| Natamba,  2014 | BMC Psychiatry | Uganda | Pregnant women | 123 | 44 | MINI | CESD | Included |
| Nogueira de Oliveira,  2014 | Epilepsy Behav | Brazil | Epilepsy patients | 126 | 35 | MINI | BDI; HADS; NDDI-E | Included |
| Prescott,  2014 | Int J Methods Psychiatr Res | USA | National Guard soldiers | 500 | NR | SCID-IV diagnosis | PHQ-9 (lifetime) | Included |
| Rathore,  2014 | Epilepsy Behav | USA | Epilepsy patients | 172 | 33 | MINI | NDDI-E; PHQ-9 | Included |
| Reme,  2014 | Spine | Norway | Chronic low back pain patients | 564 | 21 | MINI | HADS; HSCL-25 | Included |
| Schwarzbold,2014 | Rev Bras Psiquiatr | Brazil | Traumatic brain injury patients | 46 | 14 | SCID | BDI; HADS | Included |
| Stafford,  2014 | Gen Hosp Psychiatry | Australia | Women with breast or gynecologic cancer | 100 | 26 | MINI | CESD; HADS | Excluded |
| Stuart,  2014 | Compr Psychiatry | Australia | General population | 1977 | 431 | SCID-IV | Single-item screener | Included |
| Thomson,  2014 | Epilepsia | Argentina; Uruguay | Epilepsy patients | 155 | 25 | MINI | NDDI-E | Included |
| Toreki,  2014 | Midwifery | Hungary | Postpartum women | 266 | 44 | SCID | EPDS | Included |
| Tsai,  2014 | Pediatrics | China | High school students | 165 | 18 | Kiddie-SADS | PHQ-2; PHQ-9 | Included |
| Wang,  2014 | Gen Hosp Psychiatry | China | General population | 1045 | 28 | MINI | PHQ-9; SDS | Included |
| Watson,  2014 | Int J MS Care | UK | Multiple sclerosis patients | 34 | 13 | SCAN | BDI-II; HADS | Included |
| Yang,  2014 | Compr Psychiatry | China | Psycho-cardiological outpatients | 100 | 38 | MINI | HADS | Excluded |
| Asih,  2015 | Pain Pract | USA | Chronic disabling occupational musculoskeletal disorder | 542 | 343 | SCID | PHQ-9 | Included |
| Christensen,  2015 | Fam Pract | Denmark; Norway | Primary care adolescents | 294 | 33 | CIDI | HSCL-6; WHO-5 | Included |
| Couto,  2015 | J Affect Disord | Brazil | Pregnant women | 247 | 41 | MINI | BDI; EPDS | Included |
| Esiwe,  2015 | Age and Ageing | UK | Patients aged over 65 years | 118 | 26 | SCAN | GDS-15 | Included |
| Hyphantis, 2015 | J Affect Disord | Greece | Long-term medical condition patients seeking emergency services | 349 | 95 | MINI | PHQ-9 | Included |
| Mansbach,  2015 | Int Psychogeriatr | USA | Nursing home and assisted living facility patients | 224 | 122 | DSM-IV diagnosis | BADS; GDS-15 | Included |
| Martins,  2015 | J Affect Disord | Brazil | Pregnant adolescents | 807 | 143 | MINI | BDI; EPDS | Included |
| Micoulaud-Franchi,  2015 | Epilepsy Behav | France | Epilepsy patients | 116 | 33 | MINI | CESD; NDDI-E | Included |
| Patten,  2015 | Mult Scler | Canada | Multiple sclerosis patients | 152 | 20 | SCID-IV | CESD; HADS; PHQ-2; PHQ-9 | Excluded |
| Searle,  2015 | Int J Methods Psychiatr Res | Australia | Military personnel | 1798 | NR | CIDI | K-10 | Included |
| Suzuki,  2015 | PLoS One | Japan | Primary care patients | 521 | 42 | MINI | PHQ-2; PHQ-9 | Included |
| Xie,  2015 | Int Psychogeriatr | China | Patients aged 60 and older | 338 | 89 | Clinical diagnosis | GDI-SR; GDS-15 | Excluded |
| Xiong,  2015 | J Affect Disord | China | Outpatients with multiple somatic symptoms | 398 | 116 | MINI | PHQ-9 | Included |

Abbreviations: AUDADIS-IV = Alcohol Use Disorder and Associated Disabilities Interview Schedule-IV; BADS = Brief Anxiety and Depression Scale; BDI = Beck Depression Inventory; BDI-II = Beck Depression Inventory-II; BDI-FS = Beck Depression Inventory Form Screen; BDI-PC = Beck Depression Inventory for Primary Care; CESD = Center for Epidemiologic Studies Depression Scale; CIDI = Composite International Diagnostic Interview; CIS-R = Clinical Interview Schedule-revised; CUDOS = Clinically Useful Depression Outcome Scale; DASS = Depression Anxiety Stress Scales; DASS-21= Depression Anxiety Stress Scales-21; DISCs = Depression Intensity Scale Circles; DMI-10 = Depression in the Medically Ill questionnaire-10; DMI-18 = Depression in the Medically Ill questionnaire-18; DSM = Diagnostic and Statistical Manual; DT = Distress Thermometer; EPDS = Edinburgh Postnatal Depression Scale; GDS-15 = Geriatric Depression Scale-15; GDI-SR = Geriatric Depression Inventory Self Report; GHQ = General Health Questionnaire; GHQ-12 = General Health Questionnaire-12; HADS = Hospital Anxiety and Depression Scale; HADS-D = Hospital Anxiety and Depression Scale – depression subscale; HSCL-6 = Hopkins Symptom Checklist-6; HSCL-25 = Hopkins Symptom Checklist-25; IDS-SR = Inventory of Depressive Symptomology; IRIS = Indigenous Risk Impact Screen; K-6= Kessler Psychological Distress Scale-6; K-10 = Kessler Psychological Distress Scale-10; KICA-dep= Kimberley Indigenous Cognitive Assessment of Depression; Kiddie-SADS = Schedule for Affective Disorders and Schizophrenia for school-aged children; MINI = Mini-International Neuropsychiatric Interview; NDDI-E = Neurological Disorders Depression Inventory for Epilepsy; NDDIE-Youth = Neurological Disorders Depression Inventory for Epilepsy-Youth; NR = Not reported; PC-SAD = The Primary Care Screener for Affective Disorders; PDSS = Postpartum Depression Screening Scale; PHQ-2 = Patient Health Questionnaire-2; PHQ-9 = Patient Health Questionnaire-9; PRIME-MD = Primary Care Evaluation of Mental Disorders; QIDS-SR16 = Quick Inventory of Depression Symptomatology; SADS = Schedule for Affective Disorders and Schizophrenia; SCAN = Schedules for Clinical Assessment in Neuropsychiatry; SCID = Structured Clinical Interview for DSM; SDS = Self-rating depression scale; SF-12 MCS = Short Form-12 Mental Component Scale; SF-36 MCS = Short Form-36 Mental Component Scale; TLP = Time and life perception; WHO-5 = World Health Organization Wellbeing Index; ZSDS = Zung Self-Rating Depression Scale.
